# Bioremoval of Cobalt(II) from Aqueous Solution by Three Different and Resistant Fungal Biomasses

**DOI:** 10.1155/2019/8757149

**Published:** 2019-04-17

**Authors:** Juan F. Cárdenas González, Adriana S. Rodríguez Pérez, Juan M. Vargas Morales, Víctor M. Martínez Juárez, Ismael Acosta Rodríguez, Christian Michel Cuello, Gustavo Gallegos Fonseca, Milka E. Escalera Chávez, Alejandra Muñoz Morales

**Affiliations:** ^1^Unidad Académica Multidisciplinaria Zona Media, Universidad Autónoma de San Luis Potosí, Carretera San Ciro de Acosta Km. 4.0, Ejido Puente del Carmen, C.P. 79617 Río Verde, San Luis Potosí, Mexico; ^2^Universidad Autónoma de San Luis Potosí, Facultad de Ciencias Químicas, Centro de Investigación y de Estudios de Posgrado, Laboratorio de Micología Experimental, Av. Dr. Manuel Nava No. 6, Zona Universitaria, C.P. 78320 San Luis Potosí, SLP, Mexico; ^3^Área Académica de Medicina Veterinaria y Zootecnia, Instituto de Ciencias Agropecuarias, Universidad Autónoma del Estado de Hidalgo, Zona Universitaria, Rancho Universitario Km 1, C.P. 43600 Tulancingo de Bravo Hidalgo, Mexico

## Abstract

The biosorption of Co(II) on three fungal biomasses: *Paecilomyces* sp., *Penicillium* sp., and *Aspergillus niger*, was studied in this work. The fungal biomass of *Paecilomyces* sp. showed the best results, since it removes 93% at 24 h of incubation, while the biomasses of *Penicillium* sp. and *Aspergillus niger* are less efficient, since they remove the metal 77.5% and 70%, respectively, in the same time of incubation, with an optimum pH of removal for the three analyzed biomasses of 5.0 ± 0.2 at 28°C. Regarding the temperature of incubation, the most efficient biomass was that of *Paecilomyces* sp., since it removes 100%, at 50°C, while the biomasses of *Penicillium* sp. and *Aspergillus niger* remove 97.1% and 94.1%, at the same temperature, in 24 hours of incubation. On the contrary, if the concentration of the metal is increased, the removal capacity for the three analyzed biomasses decreases; if the concentration of the bioadsorbent is increased, the removal of the metal also increases. It was observed that, after 4 and 7 days of incubation, 100%, 100%, and 96.4% of Co(II) present in naturally contaminated water were removed, respectively.

## 1. Introduction

The discharge of heavy metals into aquatic ecosystems has become a matter of concern in recent decades. The contaminants of greatest concern include lead, chromium, mercury, zinc, arsenic, cadmium, copper, and cobalt, due to their toxic, carcinogenic, or mutagenic nature [1]. These toxic materials come mainly from mining operations, mineral refining, incinerator cannulas, metal treatment, fabrication of electronic equipment, paints, alloys, batteries, or pesticides [1]. The precursors commonly used for the elimination of metal ions from the effluents include chemical precipitation, coagulation-flocculation, ion exchange, reverse osmosis, and solvent extraction. These techniques, in addition to being very expensive, have some disadvantages, such as incomplete metal extraction, a large amount of reagents and energy, and the generation of toxic waste and other waste products that require special disposal [2].

Cobalt is a grayish-white metal with magnetic properties similar to those of iron and nickel; its main oxidation states are +2 and +3, but in most of the available compounds of cobalt, its value is +2. It is a relatively rare element and is produced in the earth's crust at rates ranging from 0.001-0.002%, where it is found in the form of minerals such as cobaltite (CoS_2_∙CoAs_2_), linnaeite (Co_3_S_4_), smaltite (CoAs_2_), and erythrite (3CoO∙As_2_O_5_∙8H_2_O) [3]. Its main uses are in the production of steel with special characteristics such as hardness. In the form of oxides, it is used as a catalyst in the chemical and petroleum industry, and in the form of salts, it is used as a pigment in the ceramic industry. It is also found in the wastewater coming from the nuclear plants. This metal is responsible for many bacteria, including blue-green algae that include diatoms and chrysophytes [3].

The permissible limits of cobalt in irrigation water and wastewater from livestock are 0.05 and 1.0 mg/L, respectively [4]. Acute cobalt poisoning in humans can have very serious health effects such as asthma, heart failure, and damage to the thyroid and liver [5] and can also cause mutations, and exposure to ionizing radiation is related to an increase in the risk of developing cancer [4] and decreases the growth and development in plants [6], which has increased the studies related to the removal of cobalt from wastewater. There are different physical-chemical technologies for metal removal, but due to high costs and ineffectiveness of some of them, they make bioadsorption a great alternative for the removal of trace elements [5]. In this context, biotechnology is bringing about solutions more aligned with the modern ecological demand for green processes [7]. Fungi show a capacity to absorb a great diversity of contaminants such as hydrocarbons [8], industrial wastewater [9], and metals [10] on environmentally friendly processes. Microorganisms from different genera can be promptly isolated from the environment, are fast growing, and have been showing ready adaptation to a series of challenging environmental conditions, generating many possibilities for bioremediation of cobalt, like different species of fungi: *Penicillium* sp. [11], *Paecilomyces catenlannulatus* [12], cyanobacterium *Spirulina platensis* [13], microalga *Scenedesmus dimorphus* [14], alga *Hypnea valentiae* [15], bacterium *Pseudomonas aeruginosa* SPB-1 [16], *Geobacillus thermodenitrificans* [17], and other biomasses—biochar form of *Tectona grandis* [18], chitosan grafted with maleic acid [19], and *Chrysanthemum indicum* [20]. Therefore, the objective of this study was to evaluate the removal of Co(II) in solution by the biomass of the fungi *Paecilomyces* sp., *Penicillium* sp., and *Aspergillus niger*, isolated in the presence of 500 ppm of chromium(VI).

## 2. Experimental

### 2.1. Microorganisms and Culture Conditions

**Figure 1:**
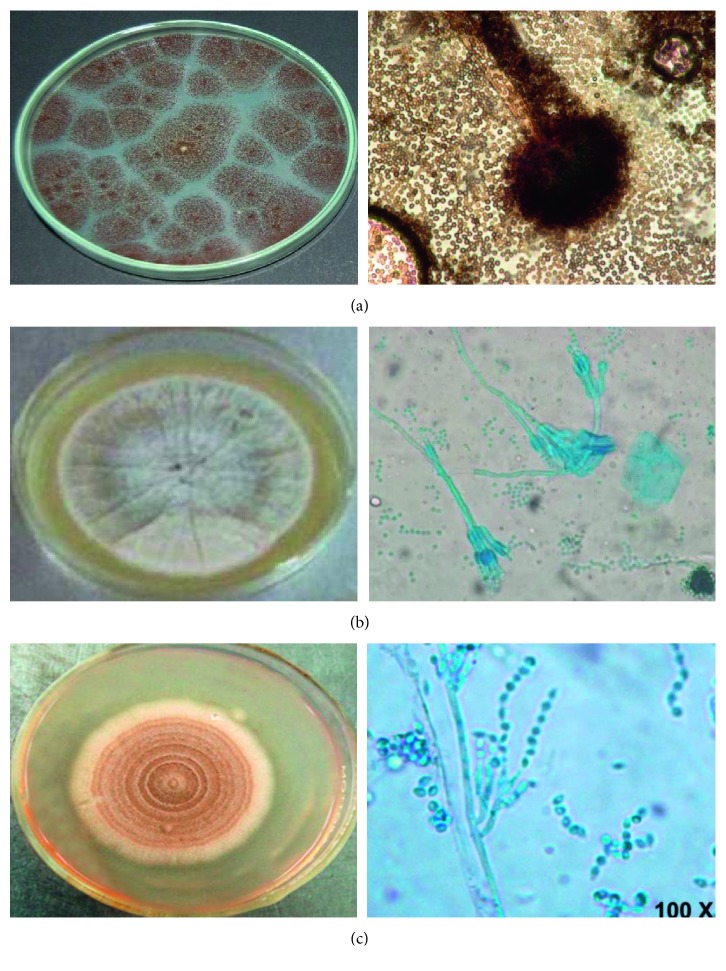
Fungal strains isolated from soil samples (100x). (a) *Aspergillus niger*; (b) *Penicillium* sp.; (c) *Paecilomyces* sp.

The biomasses of the fungi *Paecilomyces* sp., *Penicillium* sp., and *Aspergillus niger*, isolated from the air of an area close to the Faculty of Chemical Sciences of the UASLP, San Luis Potosi, S.L.P., México (the average annual temperature of 18.6°C and 1860 m above the sea level), were used in the malt agar extract. The cultures were incubated at 28°C for 7 days. The strains were identified based on their morphological structures such as color, diameter of the mycelia, and microscopic observation of formation of spores and macroconidia (Figure 1) [21].

### 2.2. Obtaining the Fungal Biomass and Cobalt(II) Solutions

The fungal biomass was obtained by inoculating a concentration of 1 × 10^6^ spores/mL in 1 L capacity Erlenmeyer flasks, which contained 600 ml of the thioglycolate broth (8 g/l); for its correct growth, the flasks were incubated at a temperature of 28°C and stirred at a constant velocity (100 rpm). The developed fungal cells were incubated for 7 days, and then they were obtained by filtering the contents of the flasks with Whatman paper No. 1, washed twice with tri-deionized water, and then dried in an oven at 80°C for 12 hours. Finally, the fungal biomass was milled and stored in an amber bottle in a refrigerator until its use. For the following analysis, a series of solutions of Co(II) with a concentration of 200 mg/L in 100 mL were prepared, which were obtained from a standard solution of 1000 mg/L previously prepared with tri-deionized water; its pH was adjusted using nitric acid and/or NaOH; another important factor was the amount of biomass added to each flask that was 1 g/100 mL for the Co(II) solutions. Samples were taken at different times, the fungal biomass was removed by centrifugation (3000 rpm/5 min), and the supernatant was analyzed to determine the concentration of the metal ion.

### 2.3. Determination of Cobalt(II)

The concentration of Co(II) in a water solution was determined by UV spectroscopy using a double-beam UV-visible spectrophotometer, Shimadzu UV-2101PC, by the method of methyl isobutyl ketone [22]. The Co(II) concentration of the samples was estimated using a calibration curve (concentration vs. absorbance) prepared with the standard concentration of Co(II) solutions. Calibration curves were prepared for each of the different pH values tested since the curves changed with pH.

## 3. Results and Discussion

### 3.1. Isolation and Identification of Fungal Strains and Their Tolerance to Co(II)

**Figure 2:**
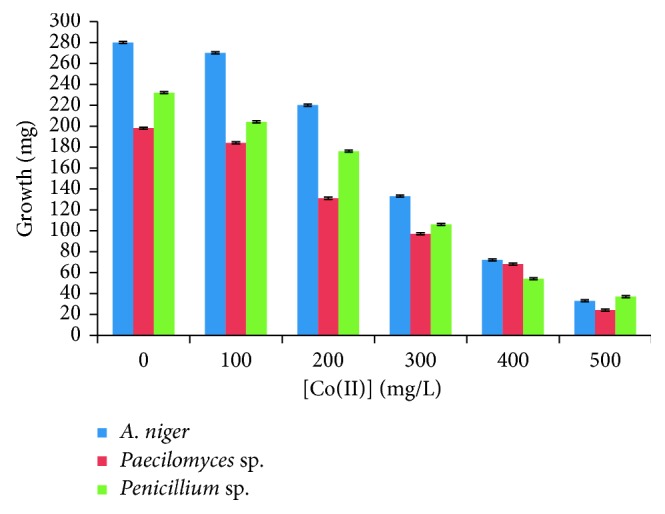
Growth in dry weight of *Aspergillus niger*, *Paecilomyces* sp., and *Penicillium* sp., with different concentrations of Co(II) (1 × 10^6^ spores/mL, 28°C, seven days of incubation, 100 rpm).

The fungal strains isolated were able to growth on LMM (Lee's minimal medium) supplemented with different concentrations of the metal (Figure 2). This indicates that these fungi developed the Co(II) tolerance and/or resistance, and they were identified by their macroscopic and microscopic characteristics [21]. The cells of the analyzed strains grew on LMM supplemented with different concentrations of Co(II) we obtained for the growth of three fungi at 500 mg/L: 33, 24, and 37 mg of dry weight, for *Aspergillus niger*, *Paecilomyces* sp., *and Penicillium* sp., respectively. It is suggested that the fungi growing at concentrations up to 500 mg/L are tolerant and/or resistant to the metal, which is similar to that reported by Acosta-Rodríguez et al. [10]; it is similar for *Candida albicans*, which grew at 300 mg/L of Co(II), for eight species of *Penicillium* isolated from Brazilian soil (tolerance between 50 and 500 *µ*g/mL); for *Pichia guilliermondii* isolated from acidic mine water in Peru, with a resistance to 400–600 mM of Co(II) [23]; and for the bacterium *Pseudomonas aeruginosa* SPB-1 (2.5 mM) [16], similar to the environmental-contaminant fungus *Penicillium* sp. IA-01 and which grew at a concentration of 500 ppm of Cr(VI) in an area near the Faculty of Chemical Sciences in San Luis Potosi, México, under the same conditions [24]. The variation in the metal tolerance might be due to the unique strategies or resistance/tolerance mechanisms exhibited by the microorganisms. Resistance to Co(II) in Gram-negative bacteria is based on the trans-envelope efflux system driven by a resistance-nodulation-cell division (RND) transporter [25].

### 3.2. The Effect of Incubation Time and pH

**Figure 3:**
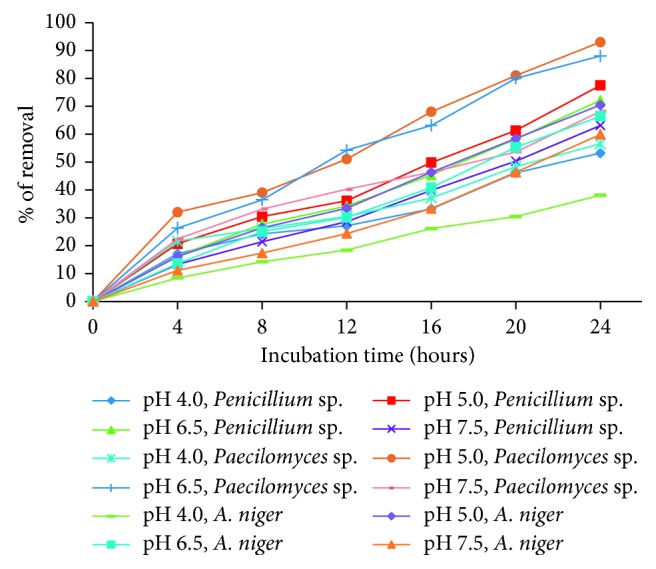
The effect of pH and incubation time on the removal of Co(II), by the different isolated fungi (200 mg/L Co(II), 28°C, 100 rpm, 5 g of fungal biomass).

Figure 3 shows the effect of contact time and pH on biosorption of Co(II) (200 mg/L) to the dried fungal biomass; it was found that the highest removal occurred in 24 h of incubation and at pH 5.0: 93%, 77.5%, and 70.4%, for *Paecilomyces* sp., *Penicillium* sp., and *Aspergillus niger*, respectively, and these results are similar to those reported by the calcium alginate of seaweed *Macrocystis pyrifera* [26], by *Cocos nucifera* L. leaf powder [27], and by *Ficus benghalensis* leaf powder [28]. But they are different from those reported by Vannela and Verma [13], who reported the maximum metal biosorption by *S. platensis* biomass which was observed at pH 6.0, with free and immobilized biomass; by *P. catenlannulatus*, as the uptake of Co(II) increases with increasing pH from 4.5 to 7.0 and at last remains at a high level at pH 7.0, due to formation of precipitates such as Co(OH)_2_(s) [12]; by alga *H. valentiae*, at a pH value of 6 [15]; and by *P. aeruginosa* SPB-1, in which the maximum adsorption of [Co(III)-EDTA] was found to be at pH 7.0 [16]. There are slightly more advanced studies with cellular fractions of the fungus *Penicillium* sp. IA-01 that indicate that the optimum pH of reduction and that of removal of Cr(VI) is 7.0, unlike the inert biomass in this study [24]. This phenomenon can be explained on the basis of the less competition between positively charged H^+^ and Co^2+^ ions for the similar functional group. As the pH rises, more ligands are exposed and the number of negatively charged groups on the adsorbent matrix probably increases, improving the removal of the cationic species [15].

### 3.3. Effect of Temperature

**Figure 4:**
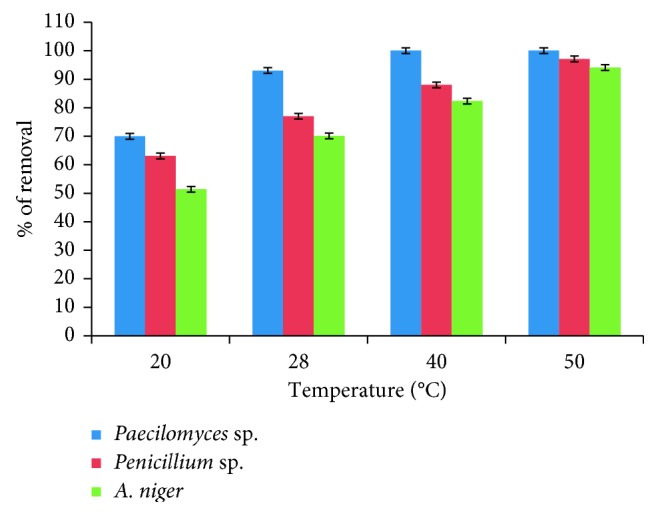
The effect of the temperature on removal of Co(II), by the different isolated fungi (200 mg/L Co(II), 100 rpm, pH 5.0, 24 h, 5 g of fungal biomass).

Figure 4 shows the effect of varying temperatures (20°C, 28°C, 40°C, and 50°C), and the maximal adsorption capacity was found at 50 ± 1°C: 100%, 97.1%, and 94.1%, for *Paecilomyces* sp., *Penicillium* sp., and *A. niger*, respectively, at 24 hours, and these results are similar to those reported for *P. catenlannulatus*, as the uptake of Co(II) increases with increasing temperature from 20 to 40°C [12], *P. aeruginosa* SPB-1 [16], *Cocos nucifera* L. leaf powder [27], and *F. benghalensis* leaf powder [28] and are different from those reported for alga *H. valentiae* [15], in which it can be seen that the maximum monolayer capacity of the adsorbent decreases from 47.44 to 46.03 mg/L by increasing the temperature from 25°C to 45°C. On the contrary, enhancement of the adsorption capacity of the fungal biomasses at higher temperatures may be attributed to the activation of the adsorbing surface and the accelerated diffusivity of the metal with the increasing temperature and increase in the mobility of metal ions [12].

### 3.4. Effect of Initial Co(II) Concentration

**Figure 5:**
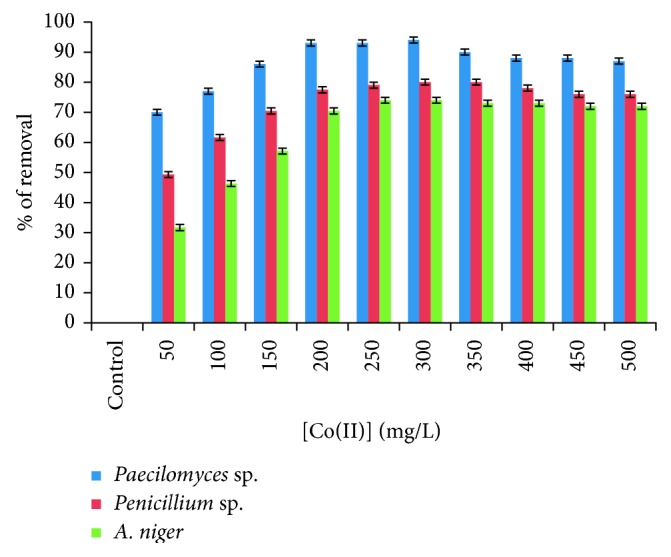
The effect of the concentration of Co(II) in solution on the removal of Co(II) by the different isolated fungi (100 rpm, 28°C, pH 5.0, 5 g of fungal biomass).

Biosorption capacities of the fungal biomasses for the Co(II) were studied as a function of the initial metal concentration between 50 and 500 mg/L (Figure 5), and the percentage of adsorption decreased, when Co(II) concentration increased from 300 mg/L for the three fungal biomasses. It has been observed that, in some genera of algae, calcium alginates of algae, and rice straw, the removal is diminished by increasing the initial concentration of Co(II) [15, 26, 29], but it is different from *S. platensis*, since increasing the initial concentration of Co(II) increases the removal of it, attaining a maximum value of 181 mg Co(II)/g at 600 mg/L concentration of Co(II) [13], for leaves of *T. grandis* (teak) tree collected from the farmlands in Vellore District, India [18]. Vilvanathan and Shanthakumar suggest that the percentage of the metal captured is directly proportional to the concentration of the metal and that it is due to the difference between forces given by the adsorption process; in *C. indicum*, it can be observed that the amount of metal ion uptake also increased from 11.7 to 15.2 mg/g with increasing Co(II) ion concentration from 25 to 75 mg/L [20]. This may be due to the increased number of competitions for the functional groups of the surface of the biomass ions [30].

### 3.5. Effect of Initial Biomass Concentration

**Figure 6:**
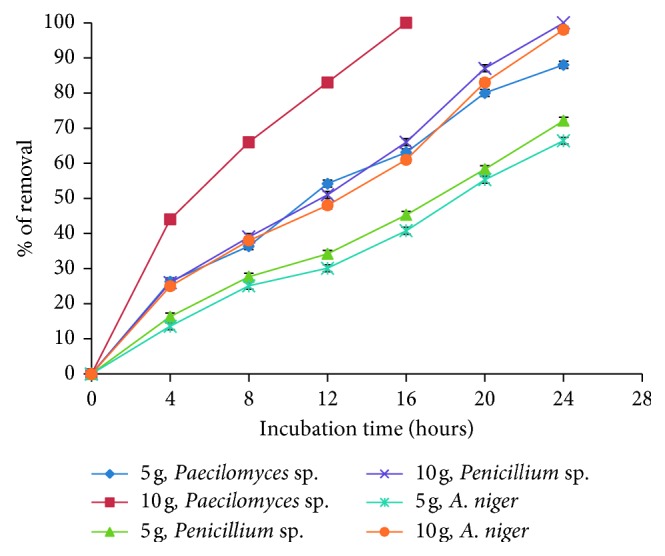
The effect of fungal biomass concentration on the removal of Co(II) (500 mg/L, 100 rpm, 28°C, pH 5.0, 24 hours).

The effect of amount of biomass on the removal capacity of Co(II) is depicted in Figure 6. If the amount of biomass is increased, the removal of the metal in the solution is also increased; when 10 g of the fungal biomass of *Paecilomyces* sp. is used instead of 5 g, it can be seen that 100% of the metal is removed in 16 hours; other filamentous fungi such as *Penicillium* sp. and *Aspergillus niger* showed excellent metal removal capacity at 24 hours with 100% and 98% removal, respectively; many authors agree with the fact that, by increasing the amount of biomass, the metal removal capacity increases because the amount of the added biosorbent determines the number of binding sites available for metal biosorption [31]. Similar results have been reported for *P. aeruginosa* SPB-1 [16], for the seaweed alga *M. pyrifera* [26], and for the adsorptive removal of Co(II) from aqueous solutions using *C. nucifera* L. [27], with nanocellulose/nanobentonite composite anchored with multicarboxyl functional group experiments, with various amounts of adsorbent ranging between 0.05 and 5.0 g/L [32].

### 3.6. Removal of Co(II) in Industrial Wastes with Fungal Biomasses

**Figure 7:**
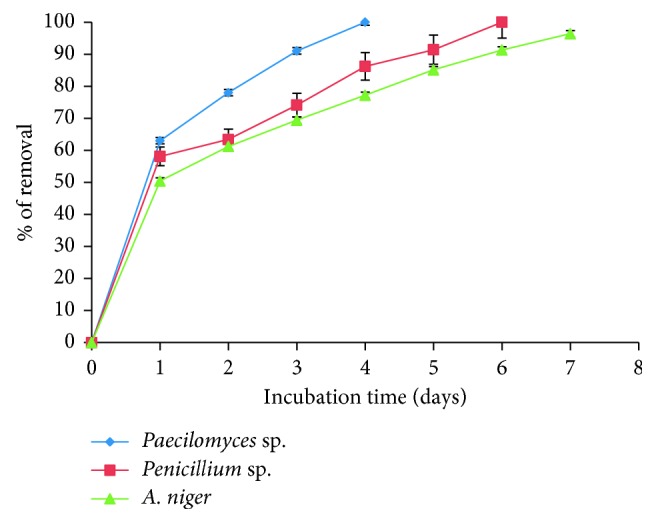
Removal of 100 mg/L of Co(II) (adjusted) from wastewater (100 rpm, 28°C, pH 5.0, 5 g of fungal biomass).

To analyze the possible application of the three fungal biomasses at the industrial level and their ability to remove Co(II) from sediments and real effluents, an aqueous solution assay was used where 5 g of the fungal biomass was used, with 100 mL of wastewater which contains 100 mg of Co(II), at 28°C and stirred at 100 rpm. The samples were taken from Tanque Tenorio (located at east of the city of San Luis Potosi, México) which was used in the seventies as a dump of industrial waste and years later also served as a wastewater dump [33]; later, these waters were used for the irrigation of crops in agriculture and as drinking troughs for animals [34]; sources of pollution were identified that generate Hg, Ba, Sr, Cd, Pb, Ag, Rb, Co, Cu, Fe, and As, and some exceed the permissible limit for human consumption [35]. In this study, we were able to observe that, after four days of incubation, 100% of Co(II) present in naturally contaminated water and soil was removed by the filamentous fungus *Paecilomyces* sp. and 96.4% by *Penicillium* sp. and *A. niger*, respectively, at seven days (Figure 7). The ability to remove by biomass is equal to or greater than that by other biomasses that have been studied, for different heavy metals, like *C. albicans* biomass to remove chromium(VI) from sediments and effluents, in which 74 and 69% of metal present in the contaminated water and soil were removed [10], and *Botryococcus braunii* biomass to remove As(III) and As(V) ions from the 50 mg/L synthetic wastewater, in which 85.22% and 88.15% of maximum removal efficiency were achieved [36]. Also, C. *tropicalis* was observed to remove 40% Cd(II) from the wastewater after 6 days and was also able to remove 78% from the wastewater after 12 days [37]; different species of the genus *Aspergillus* have the capacity to remove approximately between 20 and 50% of 100 mg/L of Hg(II) using 1 g of biomass, with a temperature of 30°C and a pH of 5.5; these data are lower than those reported in this research because mercury is more toxic and causes the inhibition of cellular glucose uptake and then cellular respiration, and therefore, there is no growth of microorganisms [38]. S*accharomyces cerevisiae* and *Torulaspora delbrueckii* decrease in 98.1, 83.0, 60.7, 60.5, and 54.2% for turbidity, sulfates, BOD, phosphates, and COD, respectively, of the tannery effluent [39], and for *S. cerevisiae*, “wild-type” (WT) parental strain BY4741 is very efficient in removing Mn(II), Cu(II), and Co(II) from synthetic effluents containing 1–2 mM cations [40].

## 4. Conclusion

The contamination by heavy metals is a serious health and ecological problem. The removal of Co(II) by three different fungal biomasses was investigated. The resistant isolated filamentous fungi have high Co(II) elimination capacity. For the determination of the removal capacity of Co(II), different aspects or operating conditions were analyzed such as incubation time, pH, initial metal ion concentration, and fungal biomass. With the data obtained, it is clearly observed that the best conditions for the elimination of Co(II) were a temperature of 50°C, a pH of 5.0, and an incubation time of 24 hours, and it was observed that, by increasing the amount of biomass to a maximum of 10 g in this study with the three resistant fungi, the metal removal capacity increased. In the case of the removal of Co(II) from natural contaminated waste such as water and soil, we could observe that the metal was removed 100% at a maximum time of four days with the fungus *Paecilomyces* sp. The easy determination of this type of biomaterial is of great ecological and economic importance for the sustainability of the ecosystems; filamentous fungi isolated *Paecilomyces* sp., *Penicillium* sp., and *Aspergillus niger* are very promising biomaterials for the removal of cobalt(II) analyzed.

## Data Availability

The images and graphics data used to support the findings of this study are included within the article; this information can also be consulted through direct contact with the authors via the following email IDs: Dr. Ismael Acosta (iacosta@uaslp.mx) and Dr. Juan Cárdenas (juan.cardenas@uaslp.mx).
